# Living prosthetic breast for promoting tissue regeneration and inhibiting tumor recurrence

**DOI:** 10.1002/btm2.10409

**Published:** 2022-09-20

**Authors:** Wenting Xu, Yu Huang, Ho‐Yin Yuen, Linli Shi, Haiqing Qian, Lijuan Cui, Mengyu Tang, Jiahui Wang, Jie Zhu, Zhirong Wang, Long Xiao, Xin Zhao, Lihong Wang

**Affiliations:** ^1^ Translational Medical Innovation Center, Zhangjiagang Traditional Chinese Medicine Hospital Affiliated to Nanjing University of Chinese Medicine Zhangjiagang Jiangsu China; ^2^ Department of Obstetrics and Gynecology The First People's Hospital of Zhangjiagang, Soochow University Zhangjiagang China; ^3^ Department of Biomedical Engineering The Hong Kong Polytechnic University Hung Hom Hong Kong

**Keywords:** Breast cancer treatment, Cell‐laden microspheres, Drug delivery, Living prosthetic breast, Regenerative medicine

## Abstract

Developing a living prosthetic breast to inhibit potential breast cancer recurrence and simultaneously promote breast reconstruction would be a promising strategy for clinical treatment of breast cancer after mastectomy. Here, a living prosthetic breast in the form of injectable gelatin methacryloyl microspheres is prepared, where they encapsulated zeolitic imidazolate framework (ZIF) nanoparticles loaded with small molecules urolithin C (Uro‐C) and adipose‐derived stem cells (ADSCs). Taking advantage of the acidic tumor microenvironment, the ZIF triggered a pH‐sensitive drug release in situ so that Uro‐C can induce tumor cell apoptosis via reactive oxygen species (ROS) generation. Meanwhile, the ADSCs proliferate in situ to promote tissue regeneration. Using such a design, our data showed that the ADSCs maintained viable and proliferate under the inhibitory effect of Uro‐C in vitro. Through ROS generation, Uro‐C also activated a suppressive tumor microenvironment in mice by both re‐polarizing M2 macrophages to M1 macrophages for elevated inflammatory responses, and increasing the ratio between CD8 and CD4 T cells for tumor recurrence inhibition, significantly promoting new adipose tissue formation. Altogether, our results demonstrate that the prepared living prosthetic breast with bifunctional properties can be a promising candidate in clinic involving tumor treatment and tissue engineering in synergy.

## 1 INTRODUCTION

As the most diagnosed and the leading causes of cancer death among women worldwide, there were roughly 2.3 million new breast cancer cases and 685,000 breast cancer deaths in 2020.^1^ Like any cancer, one of the most imminent issues the breast cancer patients face would be the risk of its recurrence after mastectomy and systemic therapy.^2^ Furthermore, the patients also face psychosocial distress postmastectomy due to the deformation of breast tissues,^3^ which leads to common procedures of prosthetic breast reconstruction right after the cancer therapy.^4^ This presents an opportunity to transform breast reconstruction as part of the clinical procedures, resulting in many incorporations of biomaterial‐based strategies, including the implantations of adipose‐derived stem cell (ADSC)‐loaded hydrogels,^5^ microsphere constructs,^6^, ^7^ and/or smart materials like photothermal scaffolds that inhibit breast cancer recurrence with both the increase in temperature and the release of tumor‐inhibiting molecules.^8^, ^9^ However, studies rarely investigate material strategies that combine both the promotion of tissue regeneration (eg, via ADSCs) and the inhibition of cancer recurrence. This could be attributed to the dose‐dependent toxicity or nonfavorable cell viability of conventional anti‐cancer small molecules (eg, doxorubicin^10^, ^11^, ^12^), which makes combining drug release in conjugation of cell delivery difficult. Urolithins, a natural intestinal metabolite which originates from ellagic acid or ellagitannin‐rich foods^13^ and is abundant in various tissues (including breast), has shown to exhibit good bioavailability and ability to suppress oncogenes, an anticancer property.^14^, ^15^, ^16^ Its intrinsic biocompatibility not only makes it a promising anticancer small drug molecule, but also a possible candidate as a cocktail therapy to inhibit tumor recurrence while combining other bioactive materials to promote breast tissue regeneration.

**SCHEME 1:**
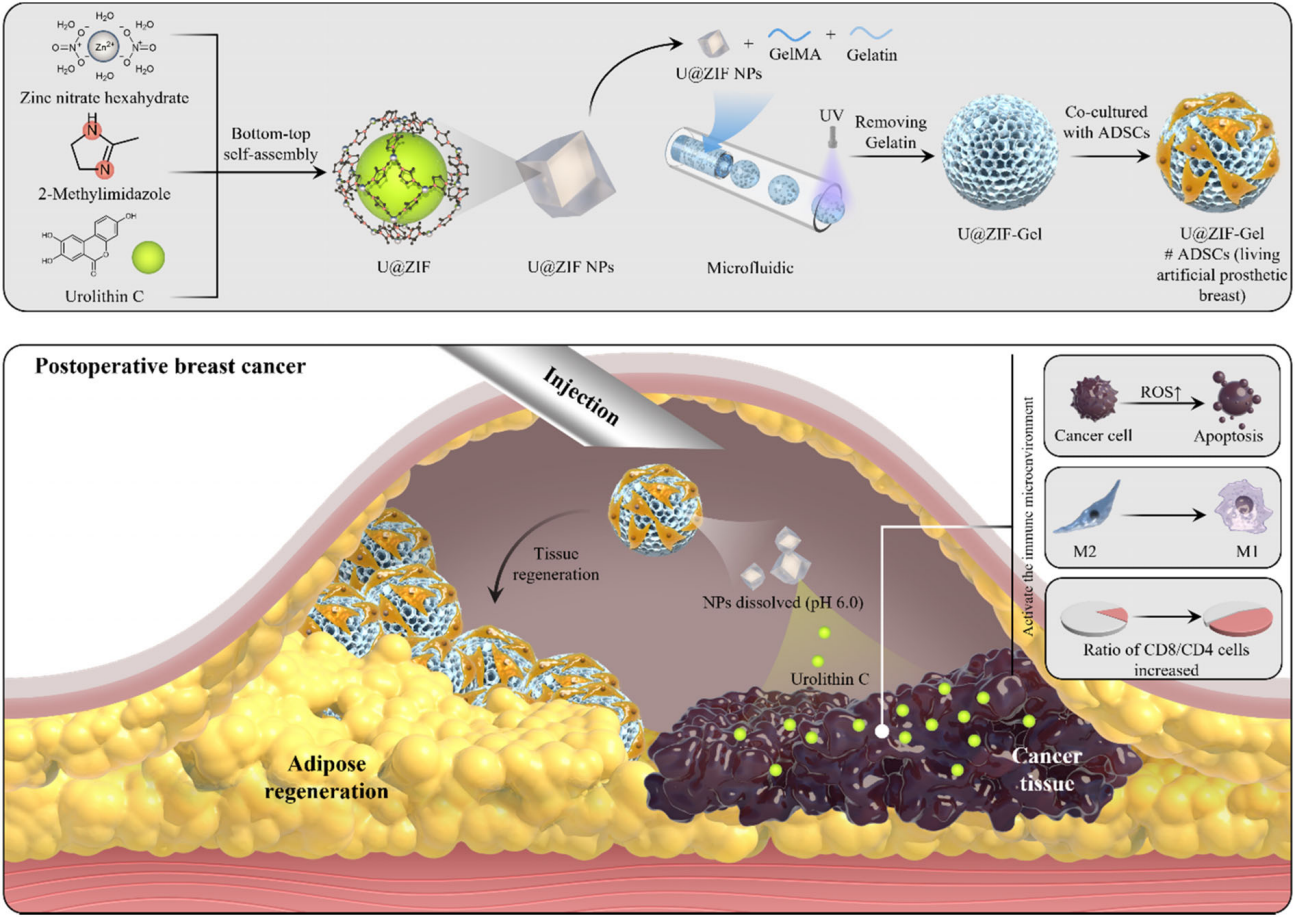
Schematic illustration of fabrication and application of living prosthetic breast (U@ZIF‐Gel#ADSCs), for inhibiting tumor recurrence and reconstructing adipose tissues for effective breast cancer treatment postmastectomy

Based on the inspiring properties of urolithins, we proposed to fabricate a pH‐sensitive living prosthetic breast for postmastectomy breast reconstruction using ADSCs for adipose regeneration, and urolithin C (Uro‐C) for pH‐sensitive drug release that reacts to the acidic tumor microenvironment (ie, pH 6.0^17^, ^18^). To achieve this, we designed the pH‐sensitive living prosthetic breast using a bottom‐up approach in the form of nanoparticle‐encapsulated microspheres (Scheme 1). Specifically, Uro‐C is first loaded into the zeolitic imidazolate frameworks (ZIFs), a type of pH‐sensitive metal‐organic framework,^19^ as a nanoparticle (denoted as U@ZIF). Then, these nanoparticles were mixed with pregel solution to form porous hydrogel microspheres. The resultant microspheres were then used to load ADSCs through coculturing, resulting in the final product—an injectable living prosthetic breast (Scheme 1). Our in vitro data show that we successfully synthesized the Uro‐C‐ and ADSC‐loaded microspheres (denoted as U@ZIF‐Gel#ADSCs), with both the Uro‐C demonstrating an initial burst and later sustained release under pH 6.0, and the ADSCs maintaining their viability and proliferation in situ. Our data also show that the release of Uro‐C led to tumor cell apoptosis due to the increased reactive oxygen species (ROS) generation. More importantly, the in vivo data demonstrate that our injectable living prosthetic breast not only exhibited an inhibitory effect on the tumor recurrence for 28 days in the C57BL/6J mice via the re‐polarization of macrophages from M2 to M1 subtypes and an increasing ratio between CD8/CD4 T cells (both indicating tumor‐killing activities at the tumor microenvironment), but also supported and enhanced adipose regeneration in the C57BL/6J mice in 2 months. All these data support that the injectable living prosthetic breast could be a promising strategy for inhibiting tumor recurrence and reconstructing adipose tissues for effective breast cancer treatment postmastectomy.

## 2 RESULTS AND DISCUSSION

### 2.1 Synthesis and characterization of U@ZIF

**FIGURE 1:**
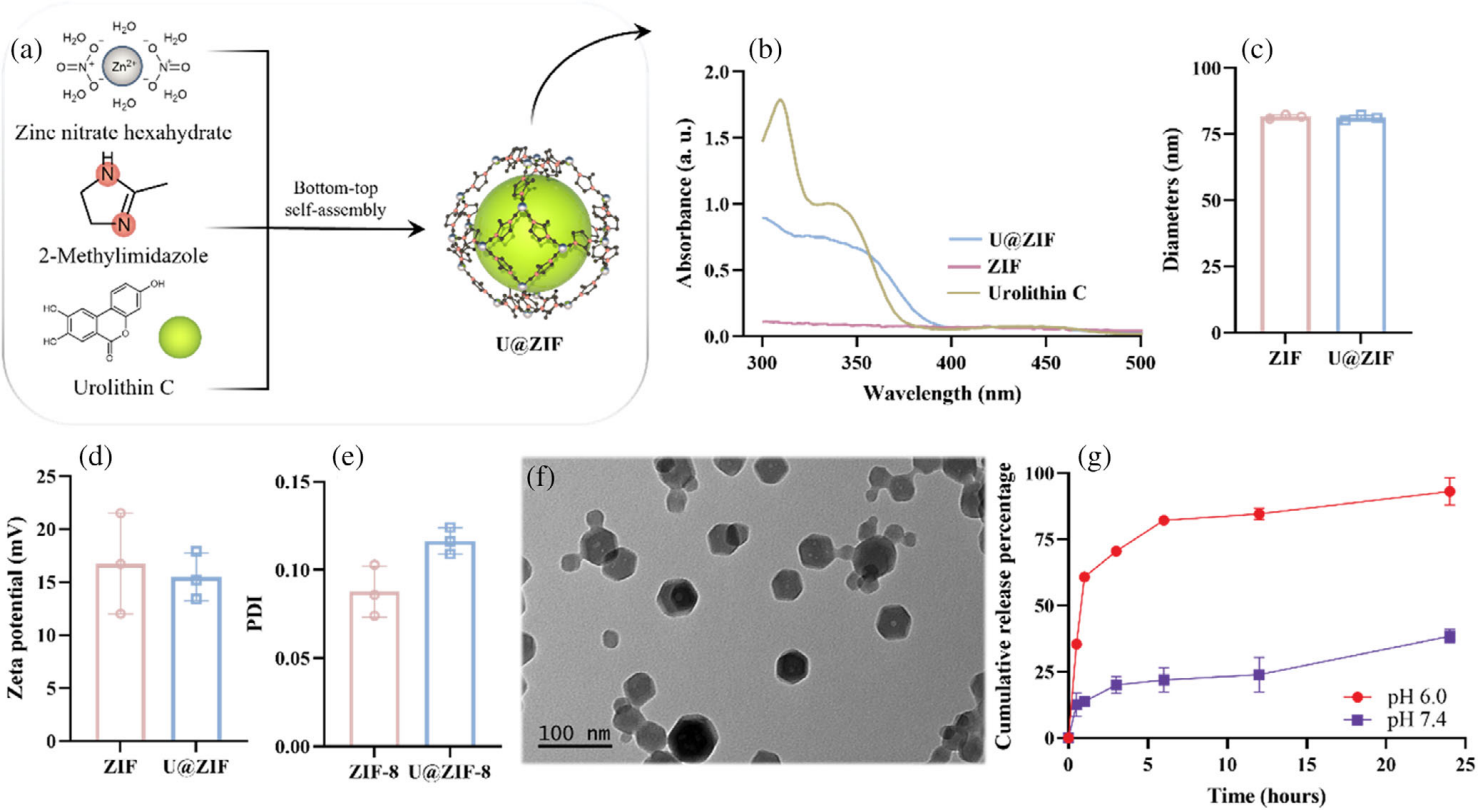
Synthesis and characterization of U@ZIF nanoparticles. (a) Schematic diagram of U@ZIF preparation. (b) UV spectra of urolithin C, ZIF, and U@ZIF. Physicochemical properties of ZIF and U@ZIF in (c) hydrodynamic diameter, (d) zeta potential, and (e) particle dispersion index. (f) Representative TEM images of U@ZIF. (g) Release behaviors of U@ZIF in phosphate buffered solution (PBS) at pH 7.4 and 6.0. The error bars are based on standard errors of the individual samples (n = 3); ANOVA with Tukey's posttest. TEM, transmission electron microscopy; UV, ultraviolet; ZIF, zeolitic imidazolate framework

The first goal in the bottom‐up design is to load Uro‐C into the pH‐sensitive zeolitic imidazolate frameworks (U@ZIF) to enable in situ tumor inhibition of our prosthetic breast. Figure 1a shows the schematic diagram of synthesis of the U@ZIF nanoparticles. Specifically, Zn^2+^ was dynamically combined with the “‐NH” on the 2‐methylimidazole to build up the ZIF frameworks. Meanwhile, the free Uro‐C was also mixed thoroughly alongside the ZIF frameworks so that the Uro‐C was encapsulated by the ZIF as the materials build up and surround each Uro‐C nanoparticle.^20^, ^21^ To explore whether the Uro‐C was successfully encapsulated by the frameworks, the ultraviolet (UV) spectra of Uro‐C, ZIF, and U@ZIF were characterized (Varioskan LUX, Thermo Scientific). Under wavelengths ranging from 300 to 500 nm (Figure 1b), the Uro‐C group exhibited significant absorbance peaks at 310 nm, but there was no obvious absorbance peak on the ZIF group, the differing absorbance of Uro‐C would allow us to identify whether Uro‐C is loaded into ZIF successfully. For the U@ZIF spectra, a decrease in absorbance peaks was observed and can be attributed to Uro‐C being encapsulated by the frameworks. The encapsulation efficiency (EE) of U@ZIF was calculated as $\frac{W_{\text{total}\;\text{urolithin}\;C}-W_{\text{unloaded urolithin}\;C}}{W_{\text{total}\;\text{urolithin}\;C}}\times 100\%$ and found to be 45.2 ± 6.7%. After we confirmed that the Uro‐C encapsulation was successful, we characterized the physicochemical properties of both ZIF and U@ZIF nanoparticles. Using dynamic light scattering (DLS), we measured their hydrodynamic diameter, zeta potential, particle dispersion index (PDI), morphology, crystal structure, and porosity. This allows us to understand more in‐depth on whether the drug‐loading affects the structure of the ZIF nanoparticles. As shown in Figure 1c‐e, there was no noticeable difference in the hydrodynamic diameter between ZIF and U@ZIF, with the *Z*‐average diameter at 81.6 ± 0.7 and 81.2 ± 1.0 nm, respectively. Additionally, both ZIF and U@ZIF exhibited positive zeta potential at 16.7 ± 4.8 and 15.5 ± 2.3 mV without significant difference. For PDI, while the relatively higher difference between U@ZIF (PDI: 0.11 ± 0.01) and ZIF (PDI: 0.08 ± 0.01) suggested that U@ZIF was less homogeneous due to the intrinsic fluctuation in EE, but it was still within the acceptable range for monodisperse particles (a PDI less than 0.1 implies monodispersity^22^). In terms of morphology, transmission electron microscopy (TEM) images show that both ZIF (Figure S1) and U@ZIF (Figure 1f) exhibited the regular hexagon shape. Altogether, our data indicate that the encapsulation of Uro‐C was successful and has caused limited influence on the physicochemical properties of U@ZIF.

After successful synthesis of U@ZIF nanoparticles, our next step was to investigate whether the pH‐triggered drug release mechanism can be activated under the two simulated physiological conditions: body fluid (pH 7.4) and tumor microenvironment (pH 6.0). The amount of Uro‐C released from U@ZIF was quantified by monitoring its absorbance at 365 nm (Varioskan LUX, Thermo Scientific). As shown in Figure 1g, the release behavior of Uro‐C was highly pH‐dependent. At physiological condition (pH 7.4), limited Uro‐C was released from the U@ZIF, arriving at 38.4 ± 2.5% after 24 hours. In contrast, 93.1 ± 5.1% Uro‐C was released from the U@ZIF in an acidic condition (pH 6.0). A burst release properties can also be observed from the Uro‐C release in acidic condition, which could be an advantageous feature to induce high stress for tumor cell death. Furthermore, a relatively lower but sustained release also retain over the remaining incubation period. These drug release profiles show that U@ZIF is a functional pH‐sensitive drug carrier to assemble the pH‐sensitive living prosthetic breast.

### 2.2 Fabrication and characterization of porous hydrogel microspheres

**FIGURE 2:**
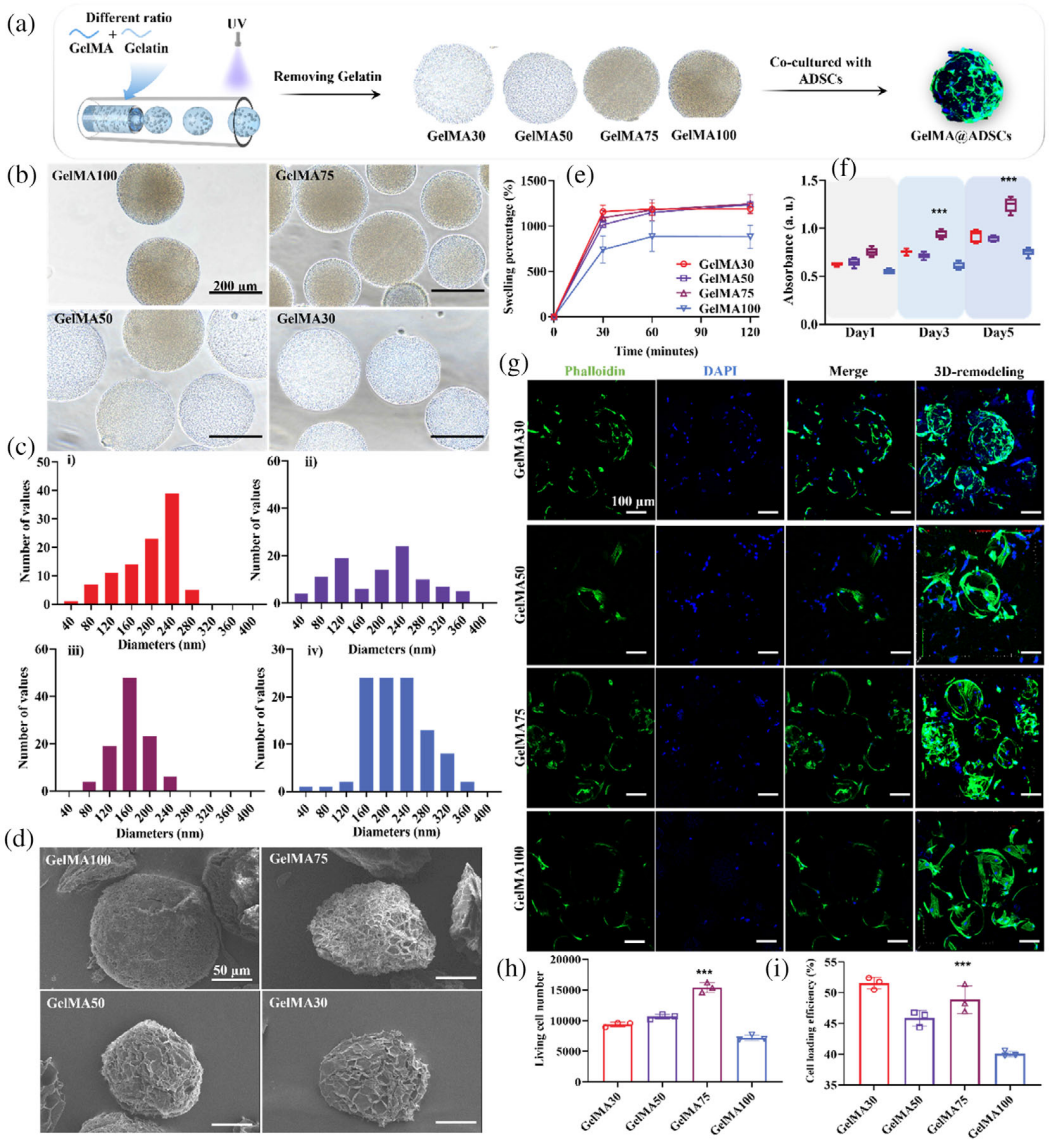
(a) Preparation of porous hydrogel microspheres with different volume ratio between GelMA and gelatin for ADSC loading. The investigation of GelMA30, GelMA50, GelMA75, and GelMA100 in (b) morphology under light microscopy. The diameter of (c) GelMA30 (ci), GelMA50 (cii), GelMA75 (ciii), and GelMA100 (civ). The morphology of GelMA30, GelMA50, GelMA75, and GelMA100 in (d) SEM and (e) their swelling percentages. (f) Quantification of ADSC proliferation in GelMA30, GelMA50, GelMA75, and GelMA100 after 1, 3, 5 days' coculture. (g) Representative confocal images for day 1 culture of ADSCs‐loaded GelMA30, GelMA50, GelMA75, and GelMA100 stained with phalloidin (green) and DAPI (blue). The day 1 samples of the ADSC‐loaded GelMA30, GelMA50, GelMA75, and GelMA100 were quantified by the (h) living cell number and (i) cell loading efficiency with viability dye using flow cytometry. The error bars are based on standard errors of the individual samples (n = 3 for (g), (h) and (i), n = 5 for the rest); ***P < .001, ANOVA with Tukey's posttest; *** indicates significance of the GelMA75 groups compared to the GelMA100 groups. ADSC, adipose‐derived stem cell; DAPI, 4′,6‐diamidino‐2‐phenylindole; GelMA, gelatin methacryloyl; SEM, scanning electron microscope

Our next step was to synthesize porous hydrogel microspheres using gelatin methacryloyl (GelMA) solutions mixed with U@ZIF nanoparticles, so that ADSCs can be loaded to enable tissue regeneration. GelMA was chosen as the microsphere material since in previous studies, it was shown that the Arg‐Gly‐Asp (RGD) sequence in GelMA can promote cell adhesion onto the surface of hydrogel microspheres.^23^, ^24^, ^25^, ^26^, ^27^, ^28^ These microspheres were prepared using a microfluidic method as depicted in Figure 2a, and their porous structure was prepared using a template sacrificing method by first mixing GelMA and gelatin at different volume ratios, and then removing the gelatin from the microspheres by washing them with deionized (DI) water in 45°C thoroughly. This creates a beneficial structure for cell infiltration. The resultant GelMA hydrogel microspheres were then cocultured with the ADSCs to prepare the GelMA@ADSCs. We denote the different volume ratios of GelMA and gelatin as follows: GelMA30 for 30% GelMA mixed with 70% gelatin; GelMA50 for 50% GelMA mixed with 50% gelatin; GelMA75 for 75% GelMA mixed with 25% gelatin; GelMA100 for 100% GelMA.

To evaluate whether the porous microspheres were synthesized successfully in accordance to the volume ratios of GelMA and gelatin, the microsphere groups GelMA30, GelMA50, GelMA75, and GelMA100 were observed under a light microscope (Figure 2b). With the increasing addition of gelatin (from GelMA100 to GelMA30), there was an increase in the transparency of the porous hydrogel microspheres due to the elimination of gelatin that led to the loose structure of the porous hydrogel microspheres. To further measure the microsphere size, 100 microspheres for each group were randomly selected and measured as shown in Figure 2c. The average diameters of GelMA30, GelMA50, GelMA75, and GelMA100 were 193 ± 54 μm, 198 ± 82 μm, 160 ± 35 μm, and 219 ± 61 μm, respectively. To further visualize the porous hydrogel microspheres, their morphology was evaluated by scanning electron microscope (SEM, Sirion 200, FEI) after freeze‐drying. In Figure 2d, enclosing spherical structures with barely any pore were observed for the GelMA100 group. With the increase in ratio of gelatin (from GelMA75 to GelMA30), the microspheres became more porous, and thus an increased surface area and porosity for cell infiltration. In addition, the GelMA30, GelMA50, and GelMA75 groups exhibited increasing swelling ratio after 30 minutes' incubation in simulated body fluid (SBF) (Figure 2e) compared to the GelMA100: the plateau swelling ratio of GelMA30, GelMA50, GelMA75, and GelMA100 were 1189 ± 127%, 1235 ± 33%, 1246 ± 225%, and 882 ± 128%, respectively, with GelMA100 having the lowest swelling ratio; this shows that the removal of gelatin allowed more room for the porous microspheres to swell.

### 2.3 Characterization of ADSC loading efficacy of porous hydrogel microspheres in vitro

Since different groups of porous hydrogel microspheres exhibited different porous structures and swelling behaviors, we then screened their cell‐loading capability by coculturing ADSCs in vitro. Figure 2f shows the cell proliferation of the ADSCs cocultured in different microsphere groups. After 1 day of coculture, there was no significant difference in the absorbance of the ADSCs cultured with GelMA30, GelMA50, GelMA75, and GelMA100. However, after 3 days and 5 days of coculture, the absorbance of ADSCs cultured with GelMA75 displayed higher values than the other three groups, indicating a more favorable cell microenvironment for proliferation. We theorize that the better performance of GelMA75 may be due to its balance in both structural integrity for cell growth and porosity for cell infiltration. To evaluate this hypothesis, we stained the day 1 samples of ADSCs in GelMA30, GelMA50, GelMA75, and GelMA100 with 4′,6‐diamidino‐2‐phenylindole (DAPI) and phalloidin to characterize its morphology (Figure 2g). After 3D reconstruction (Figure 2g), the ADSCs exhibited stretched morphology on microspheres of all groups, indicating that the ADSCs were either infiltrated into or anchored onto the surface of the microspheres. In addition, the F‐actin staining shows that the stress fibers on a single cell became progressively less stretched as the GelMA content decreased, indicating that there was lesser cell adhesion as the microspheres got looser. We also stained these ADSCs and evaluated the cells quantitatively using flow cytometry (CytoFLEX S, Beckman Coulter) for their population and viability. As shown in Figure 2h, on day 1, the GelMA75 already showed a significantly higher living cell number than all the other groups (GelMA30, GelMA50, and GelMA100), confirming its favorable microenvironment to support ADSCs. For the cell loading efficiency, it is defined the percentage of the cells loaded into the microspheres relative to the total number of cells cocultured (e.g., including cells attached onto the well plate, see Section 4.4 for further details). Here, we observed that the GelMA30 group being slightly better than the GelMA75 group. However, since GelMA75 has the greatest number of living cells (Figure 2h), this implies that apart from the GelMA75 microspheres having more living cells, there are also more living cells that are not attached to the GelMA75 microspheres, meaning that the GelMA75 group is actually more favorable for ADSC viability (consistent with the data that GelMA75 has the best proliferation in Figure 2f). Additionally, GelMA100 appeared to have the least ADSC viability, implying that a porous design is a better choice for our purpose. Considering the porosity and structure of GelMA75 that resulted in better ADSC proliferation and viability, this microsphere group was selected for the later in vitro and in vivo experiments.

### 2.4 In vitro antitumor capability of hydrogel microspheres

After evaluating the tissue regeneration capability in vitro, we systemically evaluated the ability of our living prosthetic breast to inhibit tumor recurrence. As reported, the antitumor capability of Uro‐C can be attributed to that Uro‐C increases the release of lactate dehydrogenase and lipid peroxidation malondialdehyde, which stimulates the generation of ROS and the depolarization of mitochondrial membrane, causing calcium dyshomeostasis in cells leading to apoptosis.^29^ Based on this mechanism, tumor recurrence can be inhibited by inducing apoptosis using U@ZIF to quickly release the Uro‐C under the acidic tumor microenvironment.

To produce U@ZIF‐Gel, we loaded U@ZIF into GelMA75 microspheres by mixing the nanoparticles into the pregel solution prior to microsphere fabrication. We first examined their drug release profile to characterize their pH‐sensitive property. As shown in Figure S2, pH‐triggered drug release was observed on the U‐ZIF@Gel. At physiological condition (pH 7.4), limited Uro‐C was released from the U‐ZIF@Gel, arriving at 39.4 ± 0.1% after 15 days; in contrast, 82.9 ± 1.7% of Uro‐C was released from the U‐ZIF@Gel in a tumor microenvironment (pH 6.0), demonstrating a successful synthesis of the pH‐sensitive hydrogel microsphere for Uro‐C release. We also evaluated ADSC viability in U‐ZIF@Gel by coculturing in normal growth medium for 3 days, and its resulting cell viability (Figure S3) compared to the samples without the U‐ZIF@Gel microspheres was almost the same—at around 100% viability (ie, the difference for the two groups is not statistically significant).

**FIGURE 3:**
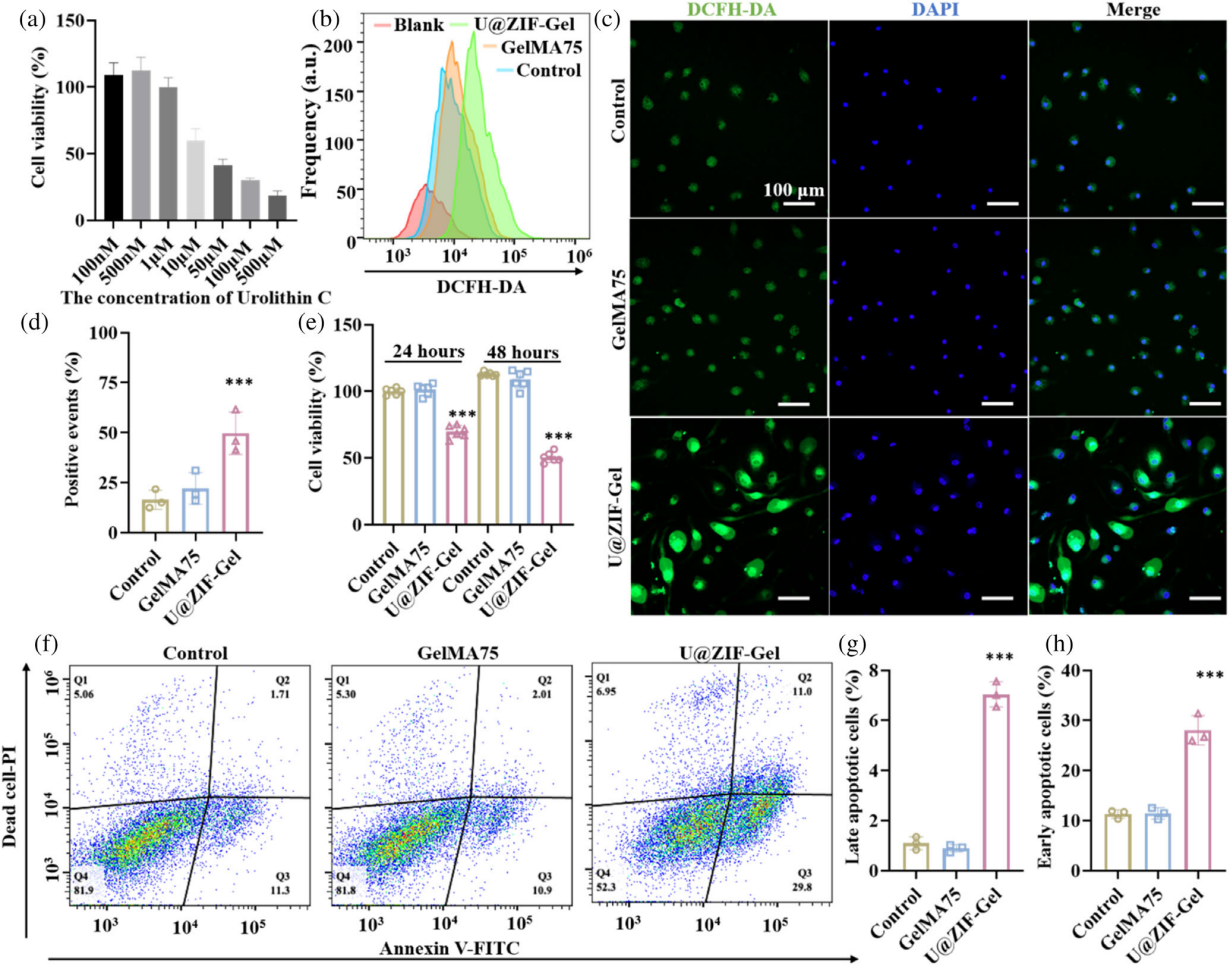
(a) Effect of concentration of urolithin C on viability of 4T1 cells. (b) Intensity of DCFH‐DA (a dye indicative of ROS level) of 4T1 cells treated with different formulations, measured by flow cytometry. (c) Representative confocal images of DCFH‐DA distribution in the 4T1 cells treated with different formulations. (d) Quantification of DCFH‐DA positive 4T1 cells. (e) Proliferation of 4T1 cells treated with different formulations after 24 hours and 48 hours of incubation. (f) Effect of different treatments on apoptosis of 4TC cells assessed by flow cytometry. Annexin V is an apoptotic marker and PI is a dead cell marker. Cells with fluorescence signals at the Q3 area imply they are in early apoptosis, whereas the Q2 area implies the late apoptosis. Quantification of (g) late apoptotic cells and (h) early apoptotic cells. Unstained 4T1 cells were denoted as Blank, 4T1 cells cultured with cell culture medium were denoted as Control, with GelMA75 denoted as GelMA75, and U@ZIF‐Gel denoted as U@ZIF‐Gel. The error bars are based on standard errors of the individual samples (n = 3 for (d) and (g), n = 6 for (e)); ANOVA with Tukey's posttest. GelMA, gelatin methacryloyl; PI, propidium iodide

After confirming the favorable properties of U‐ZIF@Gel for ADSCs, we explored the in vitro tumor inhibition capability of U‐ZIF@Gel. First, the effectiveness of direct Uro‐C incubation for the inhibition of 4T1 cells was evaluated. After 24 hours of incubation, significant inhibition effect was observed on the cell viability of 4T1 treated with 10 μM Uro‐C, where this inhibition effect on the 4T1 cells was concentration‐dependent (Figure 3a). We then investigated the inhibitory effect of U‐ZIF@Gel microspheres on the intracellular ROS level and cell apoptosis. After coculturing the 4T1 cells with either GelMA75, U‐ZIF@Gel, or blank for 4 hours, we measured the ROS level using DCFH‐DA staining (Figure 3b‐d). 4T1 cells treated with the GelMA75 exhibited a similar peak shift as 4T1 cells with blank treatment (denoted as control here), revealing that the GelMA75 microspheres alone cannot increase the ROS generation in the 4T1 cells. However, there was an obvious peak shift in the 4T1 cells treated with the U‐ZIF@Gel, indicating that the U‐ZIF@Gel can increase the ROS generation in the 4T1 cells unlike other groups. This was also visualized under the ZEISS LSM900 confocal microscope (Figure 3c). A similar trend was observed with both the control group and the GelMA75 group exhibited limited green fluorescence (indicative of ROS level of the 4T1 cells) compared to the U‐ZIF@Gel group. We further quantified the DCFH‐DA positive cells using flow cytometry, and it revealed that there was 49.6 ± 10.6% positive cells among the 4T1 cells treated with U‐ZIF@Gel, in contrast to the 16.5 ± 4.7% positive cells for control and 22.1 ± 7.9% positive cells for GelMA75. These results show that, rather than by the porous hydrogel microsphere (GelMA75) itself, the pH‐sensitive Uro‐C release of U‐ZIF@Gel is what mainly responsible to induce ROS generation.

Since U‐ZIF@Gel increased the intracellular ROS level, the cascade effect of the increased intracellular ROS was further explored by incubating 4T1 cells with U‐ZIF@Gel for 24 and 48 hours, assessed by cell counting kit‐8 (CCK‐8) (Figure 3e). After 24 hours of incubation, 4T1 cells treated with U‐ZIF@Gel exhibited decreased cell viability (69.8 ± 4.6%) compared to the 101.2 ± 4.1% for the GelMA75 group and 100 ± 2.5% for the control group. After 48 hours of incubation, the cell viability of 4T1 cells treated with U‐ZIF@Gel dropped to 50.5 ± 3.8%, in contrast with the 109.2 ± 1.5% for the GelMA75 group and 112.9 ± 6.6% for the control group. Furthermore, the mechanism behind the decreased cell viability of 4T1 cells was explored by apoptosis analysis (Figure 3f‐h) using apoptotic marker annexin V and dead cell marker propidium iodide (PI). Briefly, cells in early apoptosis presents an elevated annexin V fluorescence but a low PI fluorescence (Q3 area); and cells in late apoptosis presents an elevated fluorescence of both annexin V and PI (Q2 area). After 24 hour's incubation, there was an obvious population shift towards Q3 and Q2 in 4T1 cells treated with U‐ZIF@Gel as shown in the figures, revealing that there were more apoptotic cells in contrast with the results of GelMA75 and control (Figure 3f). Furthermore, it also revealed that 7.0 ± 0.5% of 4T1 cells were at late apoptosis stage when treated with U‐ZIF@Gel, which was significantly more than the 1.1 ± 0.3% of late apoptotic 4T1 cells in control and 0.9 ± 0.1% of late apoptotic 4T1 cells in GelMA75. As for the early apoptosis stage, there was 28.0 ± 2.9% of cells in the U‐ZIF@Gel group, 11.4 ± 1.1% for the GelMA75, and 11.3 ± 0.9% for the control. According to these results, we can conclude that U‐ZIF@Gel successfully inhibited 4T1 cells' proliferation by increasing their intracellular ROS level to induce cell apoptosis, demonstrating the efficacy of Uro‐C as a small molecule to inhibit tumor recurrence.

### 2.5 In vivo regeneration ability of hydrogel microspheres

**FIGURE 4:**
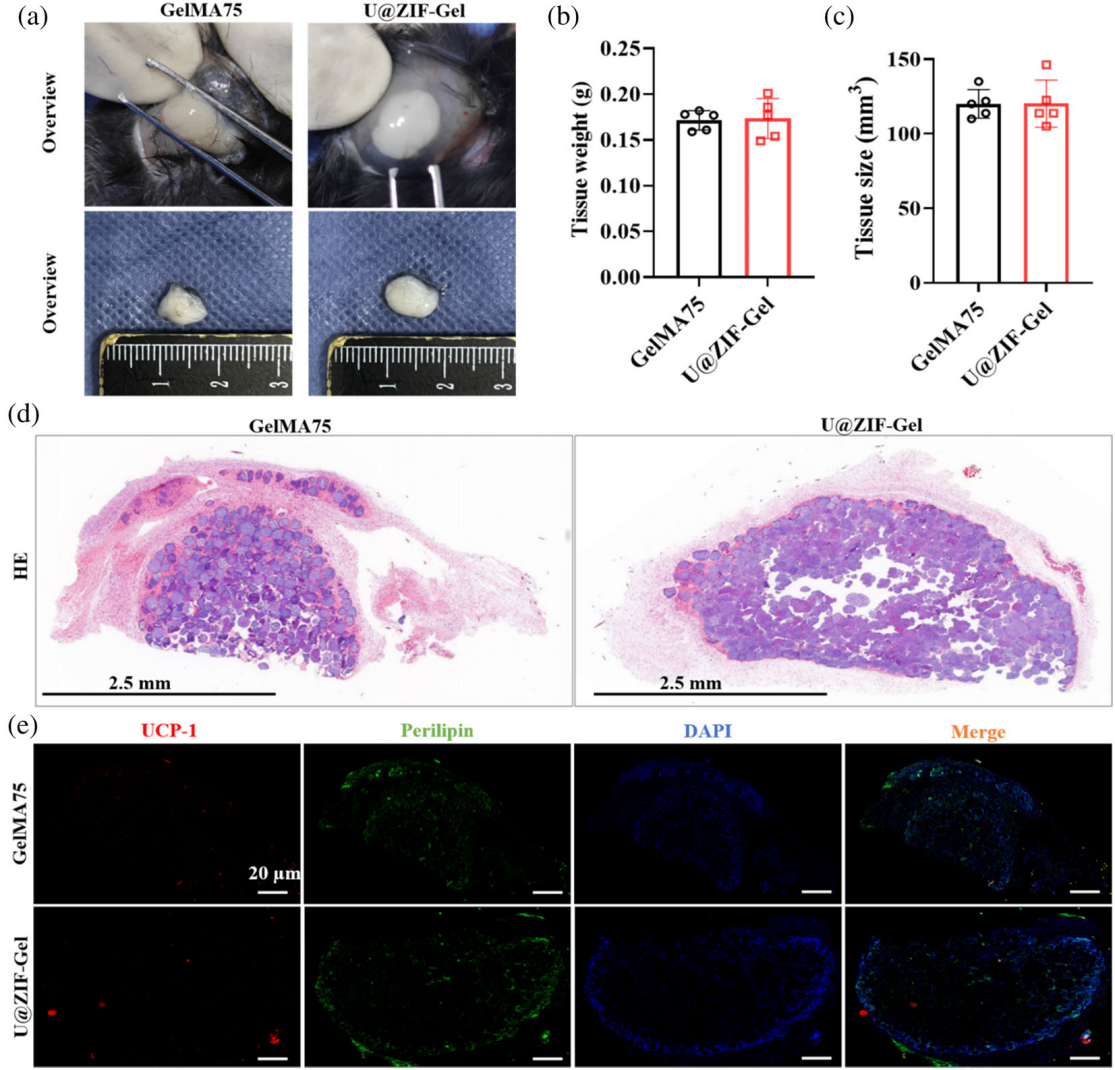
In vivo regeneration ability of hydrogel microspheres. (a) Macroscopic observation of regenerated adipose tissue. Quantification of the regenerated adipose tissue by (b) weight and (c) size. (D) Representative H&E stained images of the regenerated adipose tissue. (e) Representative immunofluorescence images of regenerated adipose tissue in UCP‐1, perilipin, DAPI, and merge. DAPI, 4′,6‐diamidino‐2‐phenylindole; UCP‐1, uncoupling protein 1

Since we demonstrated that GelMA75 carried ADSCs with good cell viability in vitro, we then investigated the in vivo regeneration effect of the microspheres by subcutaneously injecting the ADSC‐loaded GelMA75 and ADSC‐loaded U@ZIF‐Gel into the mice. Free ADSCs were also subcutaneously injected into the mice as a control group. After 2 months, we observed that there was no obvious tissue generation in the mice injected with the free ADSCs (data not shown). In comparison, mice treated with both GelMA75 and U@ZIF‐Gel exhibited regenerated tissues, as shown in Figure 4a. Quantification of the regenerated tissues by its weight and size showed that the tissue weight of the GelMA75 group was 0.171 ± 0.01 g, and the U@ZIF‐Gel group was 0.173 ± 0.02 g (Figure 4b); for tissue size, the GelMA75 group was 120.1 ± 9.6 mm^3^, and the U@ZIF‐Gel group was 120.2 ± 15.8 mm^3^ (Figure 4c). The GelMA75 and the U@ZIF‐Gel groups showed comparable results of tissue weight and size, indicating that the presence of Uro‐C in the hydrogel microspheres did not affect the regeneration ability of the hydrogel microspheres at physiological environment.

Moreover, the structure of the regenerated tissues was investigated by both hematoxylin and eosin (H&E) staining and immunofluorescence staining. As shown in Figure 4d, the regenerated tissues were located around the degraded hydrogel microspheres (ie, the site of injection) in both GelMA75 and U@ZIF‐Gel. This phenomenon indicates that the hydrogel microspheres delivered the ADSCs to the defect site for tissue regeneration. As shown in Figure 4e, similar fluorescence intensity and area for uncoupling protein 1 (UCP‐1) and perilipin can be observed in both GelMA75 and U@ZIF‐Gel, revealing that both the hydrogel microspheres with Uro‐C and without Uro‐C promoted the adipose formation without significant difference, demonstrating the tissue regenerative ability of U@ZIF‐Gel.

### 2.6 In vivo antitumor efficacy of hydrogel microspheres

**FIGURE 5:**
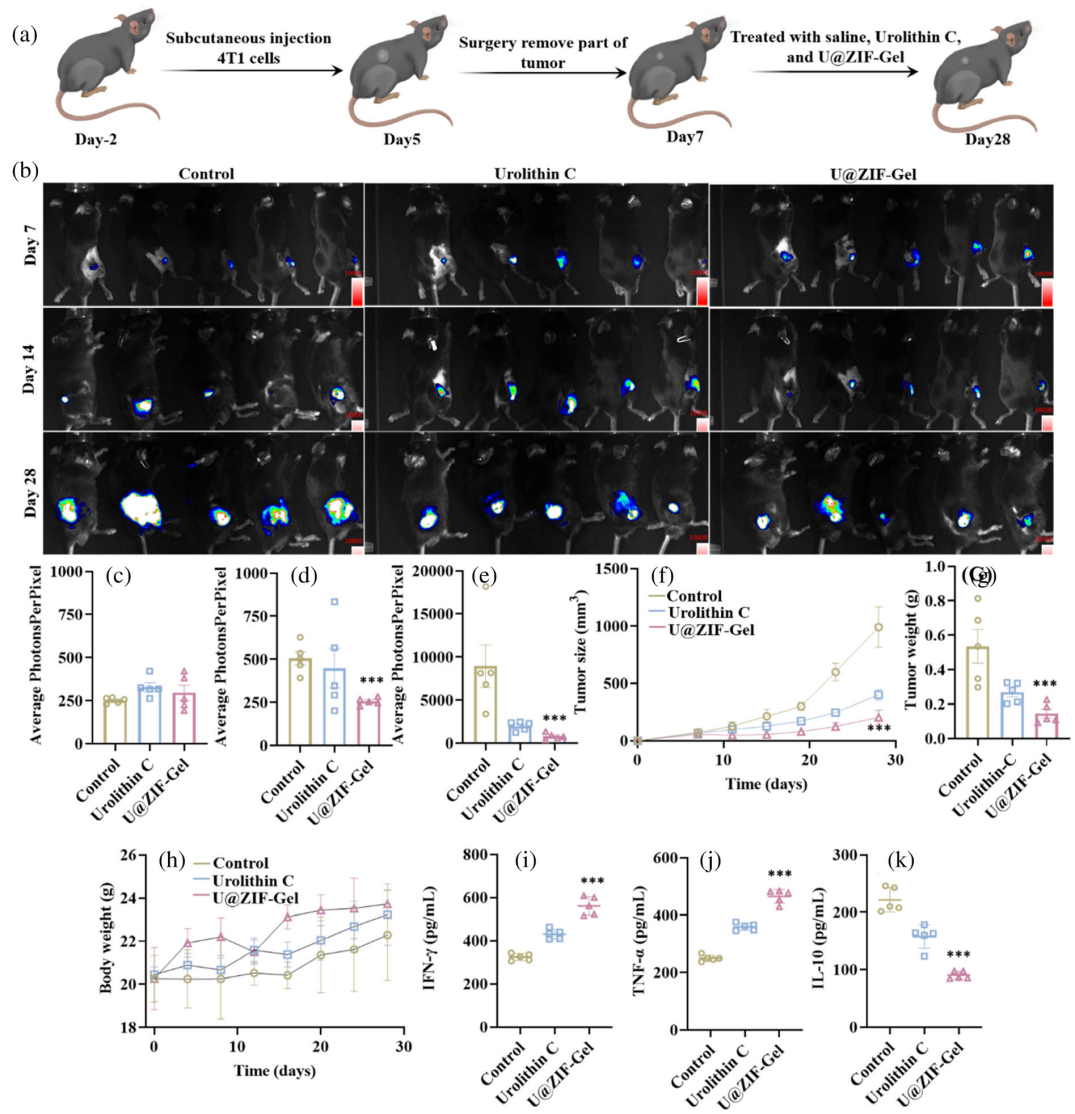
(a) Schematic diagram showing the application of U‐ZIF@Gel microspheres for tumor treatment. The 4T1‐bearing C57BL/6J mice treated with different formulations were evaluated in (b) IVIS images after 7, 14, and 28 days, and the quantified results of IVIS images after (c) 7 days, (d) 14 days, and (e) 28 days. The 4T1‐bearing C57BL/6J mice treated with different formulations were assessed in the (f) tumor size, (g) tumor weight, and (h) body weight. The ELISA results of (i) IFN‐γ, (j) IL‐10, and (k) TNF‐α in the serum of 4T1‐bearing C57BL/6J mice with different treatments. The error bars are based on standard errors of the individual samples (n = 5); ANOVA with Tukey's posttest. ELISA, enzyme linked immunosorbent assay; IFN‐γ, interferon‐γ; IL‐10, interleukin‐10; TNF‐α, tumor necrosis factor‐α

After confirming the tissue regenerative capability in vitro and in vivo, we then evaluated the tumor inhibition efficacy in vivo. To establish a tumor recurrence model, U‐ZIF@Gel was implanted into the surgery area after we removed part of the subcutaneous tumors, mimicking the situation of a postoperative breast cancer (Figure 5a). The tumor growth was monitored by an In Vivo Imaging System (IVIS) on 7, 14, and 28 days after implantation, and the mice treated with saline and free Uro‐C were denoted as control and urolithin C, respectively. As shown in Figure 5b, on day 7, there was no obvious difference in the tumor size, which indicated the successful establishment of the postoperative breast cancer model in mice, and the remaining tumor was still in development. On day 14, we observed that the tumor growth was inhibited in mice treated with U‐ZIF@Gel compared to those in the Control and Urolithin C groups. After 28 days, tumors are developed in every group, and we attribute this partially to the burst release properties of U‐ZIF@Gel (Figure 1g) that may fall short in antitumor ability for longer treatment period (albeit good performance initially), but mainly to the intentionally incomplete tumor removal that induced tumor recurrence. Nevertheless, the mice treated with U‐ZIF@Gel exhibited a smaller area than those in the control and Urolithin C group. As demonstrated by the IVIS results (Figure 5c‐e), on day 7 (Figure 5c) and day 14 (Figure 5d), there was no significant difference in the average photons per pixel among the three groups. On day 28, both U‐ZIF@Gel and Urolithin C groups displayed a lower average photons per pixel than the control group, where the U‐ZIF@Gel group was significantly lower than the Urolithin C group, suggesting an antitumor effect from the U‐ZIF@Gel group. A similar trend was observed in monitoring the tumor size and tumor weight of mice (Figure 5f,g). After 28 days' implantation (Figure 5f), the average tumor size was 991.1 ± 176.5 mm^3^ in control, 400.6 ± 45.7 mm^3^ in the Urolithin C group, and 203.5 ± 63.0 mm^3^ in the U‐ZIF@Gel group. Consistently, the average tumor weight decreased from 0.5 ± 0.2 g in the control group, 0.2 ± 0.1 g in the Urolithin C group, to 0.1 ± 0.1 g in the U‐ZIF@Gel group (Figure 5g). All these results evidenced that the U‐ZIF@Gel can enhance the antitumor efficacy of urolithin C, which could be attributed to the local and burst release of urolithin C. In practice, a complete suppression of tumors may also be achieved by repeated administration of such therapy for prolonged release, or by further adjusting the release properties of the microspheres.

Furthermore, the systemic biocompatibility of U‐ZIF@Gel was evaluated by both monitoring the body weight of mice during the whole treatment progress, and collecting the main organs for H&E staining, such as heart, liver, spleen, lung, and kidney after 28 days of treatment. As shown in Figure 5h, mice's body weight in all groups increased from day 0 to day 28, indicating the nontoxicity of the treatment. Moreover, the mice treated with U‐ZIF@Gel and urolithin C exhibited a similar tissue structure as those treated with saline (control) in all main organs (Figure S4), revealing that the application of U‐ZIF@Gel and urolithin C in the whole therapeutic progress did not cause detrimental change in the tissue structures.

Moreover, after 28 days' treatment, serum in mice was collected and analyzed for their liver functions and their immune response. To evaluate their liver function, we measured the serum level of alanine transaminase and aspartate transaminase as shown in Figure S5, and their levels were similar in all the groups (U‐ZIF@Gel, control, and Urolithin C), implying that the whole therapeutic process did not cause significant detrimental effects on the liver function of mice. For the immune system, we evaluated their level in interferon‐γ (IFN‐γ), tumor necrosis factor‐α (TNF‐α), and interleukin‐10 (IL‐10), where the former two indicate the presence of inflammation, and the latter indicate the presence of anti‐inflammation. All of which can imply the elevation of intracellular ROS in vivo. Indeed, previous studies have shown that the upregulation of these inflammatory markers and the downregulation of the anti‐inflammatory marker indicate an immune response in the target microenvironment that will induce apoptosis and hence an elevated intracellular ROS stress/signaling.^30^, ^31^, ^32^ As shown in Figure 5i‐k, mice treated with U‐ZIF@Gel exhibited up‐regulated secretion of TNF‐α and IFN‐γ and down‐regulated secretion of IL‐10, indicating the up‐regulated inflammation and activation of the immune system. Thus, it can be concluded that the U‐ZIF@Gel was activated by the tumor microenvironment and led to enhanced inflammation and tumor inhibition.

**FIGURE 6:**
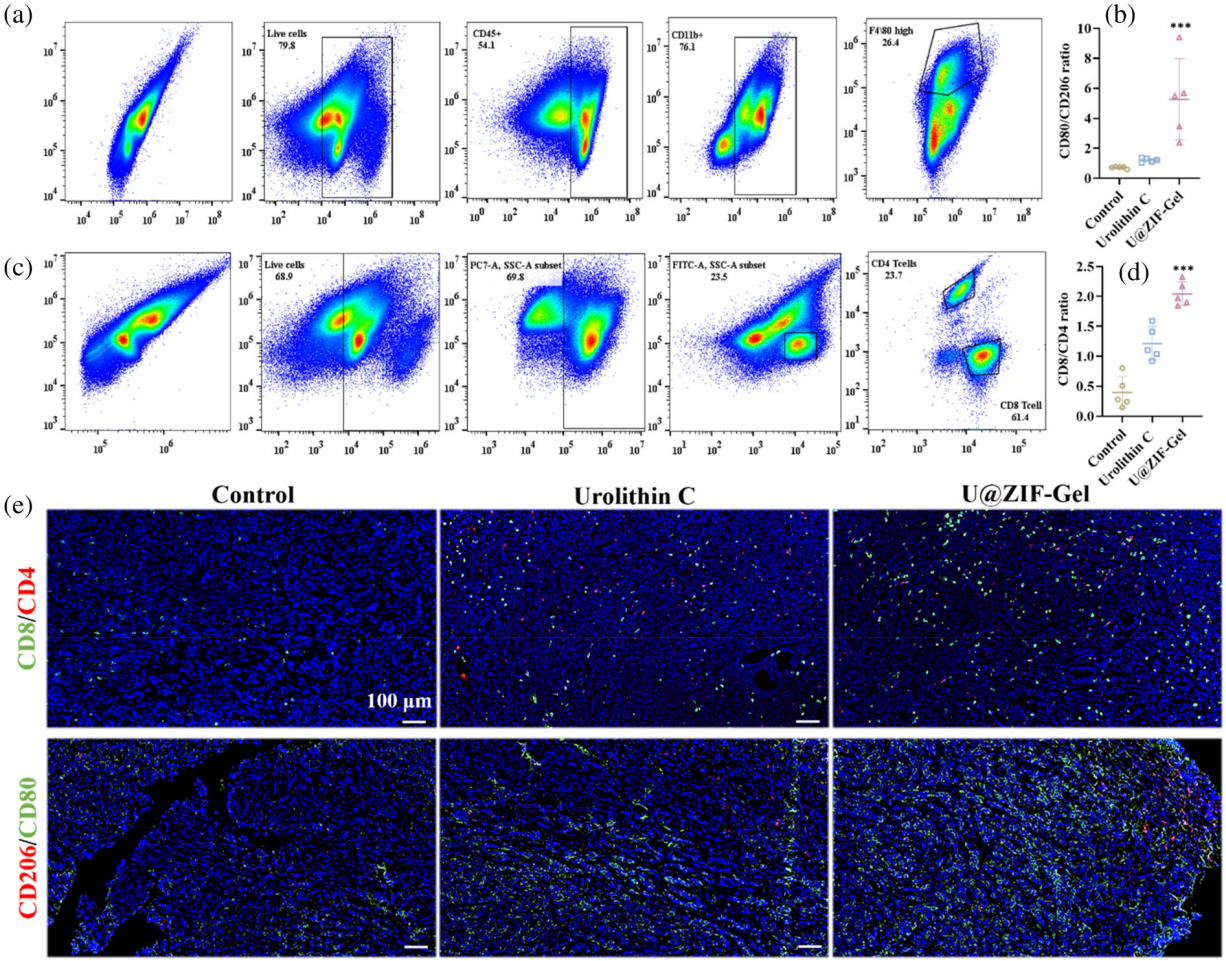
(a) The gating strategies of M1 and M2 macrophages. (b) The ratio of M1/M2 macrophages in the tumor of 4T1‐bearing C57BL/6J mice treated with different formulations. (c) The gating strategies of CD4 and CD8 T cells. (d) The ratio of CD4/CD8 T cells in the tumor of 4T1‐bearing C57BL/6J mice treated with different formulations. (e) Representative immunofluorescence images of CD8 (FITC) and CD4 (Cy3) in the tumor of 4T1‐bearing C57BL/6J mice treated with different formulations. FITC, fluorescein isothiocyanate

This conclusion was also supported by the flow cytometry analysis in the tumor tissues (Figure 6a‐d). The gating strategies for the macrophages were described in Figure 6a: (1) The living cells were selected, followed by the CD45 positive cells. (2) The CD11b positive cells were selected, followed by the high expression of F4/80 cells. (3) CD206 and CD80 positive cells were gated and quantified. First, for the CD206 and CD80 markers, they were chosen since they represent an elevated expression of M2 macrophages and M1 macrophages, respectively. Conventionally, macrophages that are polarized to M1 in tumor implies anti‐tumor activities, and to M2 for tumor promoting activities,^33^ and it has been shown clinically that a high M1/M2 ratio improves overall cancer survival (and worsen for high M2/M1 ratio).^34^, ^35^ As shown in Figure 6b, the U‐ZIF@Gel group exhibited the highest CD80/CD206 ratio (5.2 ± 2.6) compared to the 0.7 ± 0.1 for the control and 1.2 ± 0.1 for the Urolithin C group, suggesting a suppressive effect towards the tumor microenvironment. The T cell population was also investigated in Figure 6c by gating the living cells followed with the CD45 positive cells and CD3 positive cells, then gating the CD4 positive cells and CD8 positive cells, the conventional “helper” T cell marker and “killer” T cell marker respectively, in which a sufficient amount of CD4‐positive T cells is required to mobilize CD8‐positive T cells for immune responses. According to previous studies, the normal CD8/CD4 ratio was around 0.5 in healthy human and mice,^36^, ^37^ and an abnormal CD8/CD4 ratio (whether it be increasing or decreasing) can have different implications depending on the context. For example, immunosenescence could manifest an elevated CD8/CD4 ratio, but that accompanies with the loss of T cell populations.^38^, ^39^ In the case of cancer, an elevation of CD8 expression, CD4 expression, and CD8/CD4 ratio implies an inflammatory tumor‐killing microenvironment that is favorable to host survival.^40^, ^41^, ^42^ As shown in Figure 6d, the U‐ZIF@Gel group exhibited the highest CD8/CD4 ratio (2.0 ± 0.2), followed by 1.2 ± 0.3 for the Urolithin C group, and 0.4 ± 0.3 for the control group, suggesting the T cell population was upregulated to respond to the tumor development when treated with the U‐ZIF@Gel and Urolithin C group.

Moreover, the changes in the tumor microenvironment were also confirmed by immunohistochemistry (Figure 6e). For the CD8 and CD4 double staining, where the green dots indicate the CD8 positive T cells and the red dots indicate the CD4 positive T cells, it is clear that U‐ZIF@Gel had more CD8 positive T cells and CD4 positive T cells in the tumor area compared to the other two groups, indicating a more suppressed tumor microenvironment. Additionally, for the CD206 and CD80 double staining (marker for M2 and M1 macrophages respectively), where the green dots indicate the CD80 positive macrophages and the red dots indicate the CD206 positive macrophages, it was also apparent that the U‐ZIF@Gel groups exhibited more CD80 positive macrophages in the tumor area, revealing an elevated inflammatory state. All these results revealed that the U‐ZIF@Gel porous hydrogel microspheres effectively carried ADSCs and ensured the proliferation of ADSCs to promote the adipose formation in vivo, all the while inhibited tumor recurrence in situ. Altogether, our data suggest that this design could be a new generation of living prosthetic breast that simultaneously achieve tissue regeneration and antitumor efficacy.

## 3 CONCLUSION

In conclusion, we have prepared U@ZIF nanoparticle‐loaded porous hydrogel microspheres (U@ZIF‐Gel), which carried ADSCs to promote adipose regeneration and simultaneously inhibited tumor recurrence for effective prognosis of breast cancer. The porous hydrogel microspheres exhibited increased cell loading and viability, resulting in significant promotion of adipose tissue formation. Meanwhile, the tumor therapy exhibited limited side effects to the loaded ADSCs, with the pH responsive urolithin C release to inhibit the tumor tissues and the tumor cells. Moreover, the tumor recurrence can be controlled by implanting U@ZIF‐Gel for at least 28 days without any detrimental influence on the structure and function of major organs including heart, liver, spleen, lung, and kidney. Additionally, the U@ZIF‐Gel microspheres induced tumor cell apoptosis, re‐polarized M2 macrophages to M1 macrophages, and increased the ratio between CD8/CD4 T cells in tumor tissues. The U@ZIF‐Gel porous hydrogel microspheres not only integrate the advantages of controlled drug release and the capability to deliver cells to the defect site, but also take advantage of the unique physiological environment around and in tumor cells, making it a promising candidate for simultaneously inhibited tumor recurrence and promoted tissue regeneration after the surgical resection of breast tumors. We anticipate that under more advanced microsphere designs, this living prosthetic breast can be even further improved to achieve a smarter and more sustained release, bringing such an integrated therapeutic strategy closer to clinical reality.

## 4 EXPERIMENTAL SECTION

### 4.1 Synthesis and characterization of ZIFs

The urolithin C‐loaded zeolitic imidazolate frameworks (U@ZIF‐8) were prepared using a typical protocol of dissolution and centrifugation.^43^ Briefly, 4.41 mg zinc nitrate hexahydrate, 4.86 mg 2‐methylimidazole, and urolithin C was respectively dissolved into methanol. Then, these three solutions were mixed at room temperature for 10 minutes. Afterwards, the solution was centrifuged at 10,000*g* for 5 minutes, and the suspension was discarded to remove the unloaded urolithin C and unreacted chemicals (zinc nitrate hexahydrate and 2‐methylimidazole). The precipitate was collected for the following experiments.

The EE of U@ZIF‐8 was calculated as the following formulation:

$$\mathrm{EE}=\frac{W_{\text{total}\;\text{urolithin}\;C}-W_{\text{unloaded urolithin}\;C}}{W_{\text{total}\;\text{urolithin}\;C}}\times 100\%,$$

where *W* denotes the amount of the corresponding materials (either loaded or not loaded with urolithin C). Additionally, the hydrodynamic diameter, zeta potential, and PDI of U@ZIF‐8 was investigated by the DLS (Mastersizer 3000E). The morphology of U@ZIF‐8 was observed by TEM (Talos F200X S). The porous and crystal structure of U@ZIF‐8 was explored by X‐ray diffraction and Brunner Emmet Teller (BET). The UV spectrum of urolithin C was explored by the Varioskan LUX (Thermo Scientific). To characterize the release profile, the U@ZIF‐8 was incubated in the PBS at pH 6.0 and 7.4. At predetermined time points 30 minutes, 1, 3, 6, 12, and 24 hours, the suspension was collected and measured by the Varioskan LUX (Thermo Scientific) at 365 nm.

### 4.2 Preparation and characterization of porous hydrogel microspheres

#### 4.2.1 Preparation of porous hydrogel microspheres

Five percentage of GelMA solution and 2.5% gelatin solution were mixed at different volume ratio (GelMA:gelatin: 100%:0%, 75%:25%, 50%:50%, and 30%:70%, denoted as GelMA100, GelMA75, GelMA50, GelMA30) to prepare the hydrogel solution. Then, microfluidics was used to prepare the hydrogel microspheres. Specifically, paraffin containing 5% Span 80 was used as the outer phase with the speed at 600 μL/min, and hydrogel solution was used as the inner phase with the speed at 30 μL/min. The final mixture was collected at −40°C bath. Afterwards, the mixture was crosslinked under the UV light exposure (365 nm, 30 W) for 5 minutes to get the hydrogel microspheres. Then, the hydrogel microspheres were washed with ether three times to remove the paraffin, followed by washing with DI water three times at 45°C to remove ether and gelatin. Finally, the hydrogel microspheres were collected and stored at −20°C for the following experiments. The U@ZIF‐8 loaded hydrogel microspheres (U@ZIF‐Gel) were prepared similarly to the procedures above, except within the hydrogel solution, 5% of the GelMA solution was instead containing U@ZIF‐8.

#### 4.2.2 Characterization of porous hydrogel microspheres

After freeze drying, the morphology of the hydrogel microspheres was observed by SEM (Quattro). The porous structure of hydrogel microspheres was explored by the BET measurement. Additionally, the hydrogel microsphere powder was weighted and recorded as *W*_0_. Then, these hydrogel microspheres were immersed in SBF. At each time point, the hydrogel microspheres were weighted and recorded as *W*_*t*_. The swelling percentage of the hydrogel microspheres was calculated by the following formulation:

$$\text{Swelling percentage}=\frac{W_{t}}{W_{0}}\times 100\%.$$

The drug release behavior of U@ZIF‐Gel was explored by incubating U@ZIF‐Gel with PBS at pH 6.0 and 7.4. At each time points, the solution was refreshed and the concentration of urolithin C was measured by the Varioskan LUX.

### 4.3 Cell culture

ADSCs were cultured in OriCell Basal medium supplemented with 10% OriCell fetal bovine serum (FBS), and 1% penicillin‐streptomycin solution. 4T1 cells were cultured in Roswell Park Memorial Institute (RPMI)‐1640 supplemented with 10% FBS and 1% penicillin‐streptomycin solution. Cells were cultured at 37°C with 5% carbon dioxide; the culture medium was refreshed every 2 days, and cells were passaged when the cell density arrived at 90%.

### 4.4 Preparation and characterization of ADSC‐loaded hydrogel microspheres

Before culturing with cells, the hydrogel microspheres were sterilized under the Co‐60 irradiation. Afterwards, 1 mg GelMA100, GelMA75, GelMA50, and GelMA30 hydrogel microspheres were respectively weighted and added into low‐retention (low cell attachment) 96‐well plates (1 mg hydrogel microspheres for each well). ADSCs were detached, centrifuged, and resuspended in culture medium (1 × 10^6^ cells/mL). Then, 100 μL ADSC suspension was added into each well and co‐incubated with hydrogel microspheres, and cell culture medium was refreshed every 2 days. The ADSC‐loaded U@ZIF‐Gel microspheres (U@ZIF‐Gel‐ADSCs, i.e., hydrogel microspheres that also include Uro‐C loaded ZIF) were prepared similarly as described above.

After 1, 3, and 5 days' incubation, the cell viability was investigated by the CCK‐8, according to the manufacturer's instruction. 1 × 10^5^ cells cultured in the 96‐well plate was used as the negative control. The suspension was measured by the Varioskan LUX at 450 nm. After 3 days' incubation, the hydrogel microspheres were stained with phalloidin and 2‐(4‐amidinophenyl)‐6‐indolecarbamidine dihydrochloride (DAPI), and the morphology and distribution of ADSCs on the hydrogel microspheres were observed by a confocal microscope (LSM900, ZEISS). Additionally, after 3 days' incubation, the hydrogel microspheres were collected and gently washed with PBS to remove any unattached ADSCs. Then these hydrogel microspheres were incubated with 0.25% trypsin‐ethylene diamine tetraacetic acid to detach the ADSCs, followed by filtering with 40 μm cell strainer to remove the hydrogel microspheres. Finally, these detached ADSCs were stained with Zombie NIR Fixable Viability Kit and detected with a flow cytometer (CytoFLEX S, Beckman Coulter). For the day 1 culture, the living cell number and the cell loading efficiency were also quantified using flow cytometer. To measure the cell loading efficiency, we also collected ADSCs that are not loaded onto the microspheres by detaching ADSCs from the well plate and collecting ADSCs detached from the microspheres during the washing process. We denote the amount of ADSCs loaded onto the microspheres as *N*_group_, and the total amount of ADSCs collected as *N*_total_. The cell loading efficiency is then calculated as follows.

$$\text{Living cell percentage}=\frac{N_{\text{group}}}{N_{\text{total}}}\times 100\%.$$

### 4.5 In vitro antitumor efficacy of hydrogel microspheres

1 × 10^4^ 4T1 cells were seeded on the 96‐well plates with the addition of urolithin C, GelMA75, and U@ZIF‐Gel hydrogel microspheres, respectively, and 4T1 cells without any treatment were denoted as control. After 24 hours' incubation, the cell viability was evaluated by the Varioskan LUX at 450 nm, and the cell viability was calculated by the following formulation:

$$\text{Cell viability}=\frac{A_{\text{experimental groups}}}{A_{\text{control}}}\times 100\%.$$

1 × 10^5^ 4T1 cells were seeded on the 12‐well plates treated with GelMA75, U@ZIF‐Gel hydrogel microspheres, or without any treatment (denoted as control) for 4 hours, and then the cells were incubated with DCFH‐DA probe for 20 minutes. Then, cells were washed and detached, followed by analysis in the ROS by flow cytometry for fluorescein isothiocyanate (FITC) positive events. In addition, after 4 hours' treatment, cells were incubated with DCFH‐DA probe for 20 minutes followed by washing with PBS and staining with DAPI, and then the cells were observed by a confocal microscope (ZEISS). 1 × 10^5^ 4T1 cells were seeded on the 12‐well plates treated with the GelMA75, U@ZIF‐Gel hydrogel microspheres, or without any treatment (denoted as control) for 24 hours, and then the cells were detached and co‐stained with the annexin‐FITC and PI for 30 minutes, according to the manufacturer's instruction (88‐8005‐74, Thermo Fisher). Cells were then analyzed in the apoptosis by flow cytometry.

### 4.6 In vivo antitumor efficacy of hydrogel microspheres

Six‐week‐old C57BL/6J mice were purchased from JOINN Laboratories (No. 202114550, Suzhou, China). Animal experiments were approved by the Animal Ethics and Welfare Committee (AEWC) of Zhangjiagang TCM Hospital Affiliated to Nanjing University of Chinese Medicine (Approval date: 2020‐05‐20, Approval No: AEWC‐20200516). Briefly, 5 × 10^6^ 4T1 cells were subcutaneously injected into the mice under the anesthesia on day 7. After 7 days, mice were randomly divided into three groups, and half of the tumor was removed by surgery under anesthesia and analgesics on day 0. After 7 days, mice were intravenously injected with saline every 2 days (denoted as Saline), with urolithin C (0.1 mg/g) (denoted as urolithin C), and with U@ZIF‐Gel hydrogel microspheres (denoted as Gel). The tumor growth was recorded by the IVIS system (IVIS Lumina III, PerkinElmer) on days 7, 14, and 28. The tumor volume and body weight of mice was also recorded, and the tumor volume was calculated by the following formulation: $V=\frac{\text{Length}\times {\text{Width}}^{2}}{2}$.

On day 28, mice were euthanized by CO_2_. Major organs, such as heart, liver, spleen, lung, and kidney, were collected and processed with H&E staining. Blood was collected with the addition of heparin sodium, and serum was isolated by the centrifugation (900*g*) at 4°C for 15 minutes. Then, the concentration of IL‐10, TNF‐α, and IFN‐γ in the serum was evaluated by the enzyme linked immunosorbent assay.

Tumor was first isolated and weighted, then cut into two parts. Half of the tumor was processed with the immunofluorescence staining (CD80, CD206, CD4, and CD8). The remaining tumor was digested into the single cell suspension. Specifically, tumor was cut into small pieces and incubated in RPMI‐1640 medium containing 1 mg/mL collagenase type I, 0.1 mg/mL deoxyribonuclease, and 2 U/mL hyaluronidase for 30 minutes with constant vibration. Afterwards, the tumor tissues were smashed and filtered with 70 μm cell strainer to prepare the single cell suspension. The cell suspension was first blocked with the CD16/32 for 15 minutes at 4°C, followed by staining with Zombie NIR Fixable Viability Kit for 15 minutes at 4°C. Then cells were respectively stained with CD45, CD11b, F4/80, CD80, and CD206 for the macrophage subtype, and CD45, CD3, CD8, CD4, CD25, and FOXP3 for the T cell subtypes.

### 4.7 In vivo tissue regeneration of hydrogel microspheres

ADSCs were respectively co‐cultured with GelMA75 and U@ZIF‐Gel hydrogel microspheres for 7 days. Then, these ADSC‐loaded microspheres were subcutaneously injected into the right back of C57BL/6J mice. Mice subcutaneously injected with free ADSCs were regarded as the control group. After 2 months, the tissues around the right back of C57BL/6J mice were collected and weighted. Afterwards, the tissues were processed with H&E staining and immunofluorescence staining (uncoupling protein 1, UCP1‐Cy3, and Perilipin‐FITC).

### 4.8 Statistical analysis

All results are representative of data generated in three independent experiments unless otherwise stated. All numerical values were expressed as the mean ± SD. For multiple comparison, statistical analysis was performed using one‐way ANOVA followed by a Bonferroni posttest. For individual comparison, statistical analysis was performed using two‐tailed *t*‐test. Data analysis was performed using SPSS 22.0 software and considered statistically significant at *P* < .05.

## AUTHOR CONTRIBUTIONS

**Wenting Xu:** Methodology (equal); writing – original draft (equal). **Yu Huang:** Data curation (equal); formal analysis (equal); methodology (equal). **Ho‐Yin Yuen:** Writing – original draft (equal); writing – review and editing (equal). **Linli Shi:** Data curation (equal); formal analysis (equal). **Haiqing Qian:** Formal analysis (equal); software (equal). **Lijuan Cui:** Validation (equal); visualization (equal). **Mengyu Tang:** Investigation (equal); visualization (equal). **Jiahui Wang:** Investigation (equal); methodology (equal). **Jie Zhu:** Methodology (equal). **Zhirong Wang:** Funding acquisition (equal); project administration (equal); supervision (equal). **Xin Zhao:** Resources (equal); writing – original draft (equal); writing – review and editing (equal).

### PEER REVIEW

The peer review history for this article is available at https://publons.com/publon/10.1002/btm2.10409.

## Supporting information


**Figure S1.** Representative TEM images of ZIF
**Figure S2.** Drug release behaviors of U@ZIF‐Gel at pH 7.4 and pH 6.0. The error bars are based on standard errors of the individual samples (n = 3); ANOVA with Tukey's posttest
**Figure S3.** Cell viability of ADSCs cultured with U‐ZIF@Gel and with medium (NC). The error bars are based on standard errors of the individual samples (n = 5); ANOVA with Tukey's posttest
**Figure S4.** Representative H&E stained images of the major organs, heart, liver, spleen, lung, and kidney, in mice treated with saline (Control), urolithin C, and U@ZIF‐Gel
**Figure S5.** Serum levels of ALT and AST in mice treated with U‐ZIF@Gel, control, and urolithin C. The error bars are based on standard errors of the individual samples (n = 5); ANOVA with Tukey's posttest

## Data Availability

Data available on request from the authors.

## References

[1] Lei S , Zheng R , Zhang S , et al. Global patterns of breast cancer incidence and mortality: a population‐based cancer registry data analysis from 2000 to 2020. Cancer Commun. 2021;41(11):1183‐1194. doi:10.1002/cac2.12207 PMC8626596 34399040

[2] Crowe JP Jr , Gordon NH , Antunez AR , Shenk RR , Hubay CA , Shuck JM . Local‐regional breast cancer recurrence following mastectomy. Arch Surg. 1991;126(4):429‐432. doi:10.1001/archsurg.1991.01410280027002 2009056

[3] Weitzner MA , Meyers CA , Stuebing KK , Saleeba AK . Relationship between quality of life and mood in long‐term survivors of breast cancer treated with mastectomy. Support Care Cancer. 1997;5(3):241‐248. doi:10.1007/s005200050067 9176972

[4] McCarthy CM , Pusic AL , Sclafani L , et al. Breast cancer recurrence following prosthetic, postmastectomy reconstruction: incidence, detection, and treatment. Plast Reconstr Surg. 2008;121(2):381‐388. doi:10.1097/01.prs.0000298316.74743.dd 18300953

[5] Yang X , Gao L , Wei Y , et al. Photothermal hydrogel platform for prevention of post‐surgical tumor recurrence and improving breast reconstruction. J Nanobiotechnology. 2021;19(1):307. doi:10.1186/s12951-021-01041-w 34620160 PMC8499550

[6] O'Halloran N , Courtney D , Kerin MJ , Lowery AJ . Adipose‐derived stem cells in novel approaches to breast reconstruction: their suitability for tissue engineering and oncological safety. Breast Cancer Basic Clin Res. 2017;11:1178223417726777. doi:10.1177/1178223417726777 PMC5562338 29104428

[7] Yang IH , Chen YS , Li JJ , et al. The development of laminin‐alginate microspheres encapsulated with Ginsenoside Rg1 and ADSCs for breast reconstruction after lumpectomy. Bioact Mater. 2021;6(6):1699‐1710. doi:10.1016/j.bioactmat.2020.11.029 33313449 PMC7710511

[8] He C , Yu L , Yao H , Chen Y , Hao Y . Combinatorial photothermal 3D‐printing scaffold and checkpoint blockade inhibits growth/metastasis of breast cancer to bone and accelerates osteogenesis. Adv Funct Mater. 2021;31(10):2006214. doi:10.1002/adfm.202006214

[9] Wu Y , Wang H , Gao F , Xu Z , Dai F , Liu W . An injectable supramolecular polymer nanocomposite hydrogel for prevention of breast cancer recurrence with theranostic and mammoplastic functions. Adv Funct Mater. 2018;28(21):1801000. doi:10.1002/adfm.201801000

[10] Longhi A , Ferrari S , Bacci G , Specchia S . Long‐term follow‐up of patients with doxorubicin‐induced cardiac toxicity after chemotherapy for osteosarcoma. Anticancer Drugs. 2007;18(6):737‐744. doi:10.1097/CAD.0b013e32803d36fe 17762406

[11] Shafei A , El‐Bakly W , Sobhy A , et al. A review on the efficacy and toxicity of different doxorubicin nanoparticles for targeted therapy in metastatic breast cancer. Biomed Pharmacother. 2017;95:1209‐1218. doi:10.1016/j.biopha.2017.09.059 28931213

[12] Zubizarreta ME , Xiao S . Bioengineering models of female reproduction. Biodes Manuf. 2020;3(3):237‐251. doi:10.1007/s42242-020-00082-8 32774987 PMC7413245

[13] Espín JC , Larrosa M , García‐Conesa MT , Tomás‐Barberán F . Biological significance of urolithins, the gut microbial ellagic acid‐derived metabolites: the evidence so far. Evid Based Complement Alternat Med. 2013;2013:e270418. doi:10.1155/2013/270418 PMC3679724 23781257

[14] Al‐Harbi SA , Abdulrahman AO , Zamzami MA , Khan MI . Urolithins: the gut based polyphenol metabolites of ellagitannins in cancer prevention, a review. Front Nutr. 2021;8:647582. doi:10.3389/fnut.2021.647582 34164422 PMC8215145

[15] Senobari Z , Karimi G , Jamialahmadi K . Ellagitannins, promising pharmacological agents for the treatment of cancer stem cells. Phytother Res. 2022;36(1):231‐242. doi:10.1002/ptr.7307 34697838

[16] Stanisławska IJ , Piwowarski JP , Granica S , Kiss AK . The effects of urolithins on the response of prostate cancer cells to non‐steroidal antiandrogen bicalutamide. Phytomedicine. 2018;46:176‐183. doi:10.1016/j.phymed.2018.03.054 30097116

[17] Du J , Lane LA , Nie S . Stimuli‐responsive nanoparticles for targeting the tumor microenvironment. J Control Release. 2015;219:205‐214. doi:10.1016/j.jconrel.2015.08.050 26341694 PMC4656063

[18] Qin J , Zhu Y , Zheng D , Zhao Q . pH‐sensitive polymeric nanocarriers for antitumor biotherapeutic molecules targeting delivery. Biodes Manuf. 2021;4(3):612‐626. doi:10.1007/s42242-020-00105-4

[19] Sun CY , Qin C , Wang XL , et al. Zeolitic imidazolate framework‐8 as efficient pH‐sensitive drug delivery vehicle. Dalton Trans. 2012;41(23):6906‐6909. doi:10.1039/C2DT30357D 22580798

[20] Lu G , Li S , Guo Z , et al. Imparting functionality to a metal–organic framework material by controlled nanoparticle encapsulation. Nat Chem. 2012;4(4):310‐316. doi:10.1038/nchem.1272 22437717

[21] Wang W , Pan X , Yang H , et al. Bioactive metal–organic frameworks with specific metal–nitrogen (M–N) active sites for efficient sonodynamic tumor therapy. ACS Nano. 2021;15(12):20003‐20012. doi:10.1021/acsnano.1c07547 34860487

[22] Raval N , Maheshwari R , Kalyane D , Youngren‐Ortiz SR , Chougule MB , Tekade RK . Importance of physicochemical characterization of nanoparticles in pharmaceutical product development. In: Tekade RK , ed. Basic Fundamentals of Drug Delivery (Advances in Pharmaceutical Product Development and Research). Academic Press; 2019:369‐400. doi:10.1016/B978-0-12-817909-3.00010-8 Chapter 10.

[23] Zhao X , Liu S , Yildirimer L , et al. Injectable stem cell‐laden photocrosslinkable microspheres fabricated using microfluidics for rapid generation of osteogenic tissue constructs. Adv Funct Mater. 2016;26(17):2809‐2819. doi:10.1002/adfm.201504943

[24] Zhao X , Lang Q , Yildirimer L , et al. Photocrosslinkable gelatin hydrogel for epidermal tissue engineering. Adv Healthc Mater. 2016;5(1):108‐118. doi:10.1002/adhm.201500005 25880725 PMC4608855

[25] Zhao X , Sun X , Yildirimer L , et al. Cell infiltrative hydrogel fibrous scaffolds for accelerated wound healing. Acta Biomater. 2017;49:66‐77. doi:10.1016/j.actbio.2016.11.017 27826004 PMC5296408

[26] Yang Y , Xu T , Zhang Q , Piao Y , Bei HP , Zhao X . Biomimetic, stiff, and adhesive periosteum with osteogenic–angiogenic coupling effect for bone regeneration. Small. 2021;17(14):2006598. doi:10.1002/smll.202006598 33705605

[27] Liang X , Xie L , Zhang Q , et al. Gelatin methacryloyl‐alginate core‐shell microcapsules as efficient delivery platforms for prevascularized microtissues in endodontic regeneration. Acta Biomater. 2022;144:242‐257. doi:10.1016/j.actbio.2022.03.045 35364321

[28] Wu J , Li G , Ye T , et al. Stem cell‐laden injectable hydrogel microspheres for cancellous bone regeneration. Chem Eng J. 2020;393:124715. doi:10.1016/j.cej.2020.124715

[29] Yin P , Zhang J , Yan L , et al. Urolithin C, a gut metabolite of ellagic acid, induces apoptosis in PC12 cells through a mitochondria‐mediated pathway. RSC Adv. 2017;7(28):17254‐17263. doi:10.1039/C7RA01548H

[30] Thaxton JE , Romero R , Sharma S . TLR9 activation coupled to IL‐10 deficiency induces adverse pregnancy outcomes. J Immunol. 2009;183(2):1144‐1154. doi:10.4049/jimmunol.0900788 19561095 PMC2785500

[31] Zhang B , Zeng M , Li M , et al. Protopine protects mice against LPS‐induced acute kidney injury by inhibiting apoptosis and inflammation via the TLR4 signaling pathway. Molecules. 2020;25(1):15. doi:10.3390/molecules25010015 PMC6982873 31861525

[32] Sauer H , Wartenberg M , Hescheler J . Reactive oxygen species as intracellular messengers during cell growth and differentiation. Cell Physiol Biochem. 2001;11(4):173‐186. doi:10.1159/000047804 11509825

[33] Oshi M , Tokumaru Y , Asaoka M , et al. M1 macrophage and M1/M2 ratio defined by transcriptomic signatures resemble only part of their conventional clinical characteristics in breast cancer. Sci Rep. 2020;10(1):16554. doi:10.1038/s41598-020-73624-w 33024179 PMC7538579

[34] Nowak M , Klink M . The role of tumor‐associated macrophages in the progression and chemoresistance of ovarian cancer. Cell. 2020;9(5):1299. doi:10.3390/cells9051299 PMC7290435 32456078

[35] Zhang M , He Y , Sun X , et al. A high M1/M2 ratio of tumor‐associated macrophages is associated with extended survival in ovarian cancer patients. J Ovarian Res. 2014;7(1):19. doi:10.1186/1757-2215-7-19 24507759 PMC3939626

[36] Amadori A , Zamarchi R , De Silvestro G , et al. Genetic control of the CD4/CD8 T‐cell ratio in humans. Nat Med. 1995;1(12):1279‐1283. doi:10.1038/nm1295-1279 7489409

[37] Goldman JP , Blundell MP , Lopes L , Kinnon C , DI Santo JP , Thrasher AJ . Enhanced human cell engraftment in mice deficient in RAG2 and the common cytokine receptor γ chain. Br J Haematol. 1998;103(2):335‐342. doi:10.1046/j.1365-2141.1998.00980.x 9827902

[38] McBride JA , Striker R . Imbalance in the game of T cells: what can the CD4/CD8 T‐cell ratio tell us about HIV and health? PLoS Pathog. 2017;13(11):e1006624. doi:10.1371/journal.ppat.1006624 29095912 PMC5667733

[39] Noppert GA , Stebbins RC , Dowd JB , Aiello AE . Sociodemographic differences in population‐level immunosenescence in older age. medRxiv. 2022. doi:10.1101/2022.03.05.22271952

[40] Hadrup S , Donia M , Thor Straten P . Effector CD4 and CD8 T cells and their role in the tumor microenvironment. Cancer Microenviron. 2013;6(2):123‐133. doi:10.1007/s12307-012-0127-6 23242673 PMC3717059

[41] Ruffell B , Au A , Rugo HS , Esserman LJ , Hwang ES , Coussens LM . Leukocyte composition of human breast cancer. Proc Natl Acad Sci. 2012;109(8):2796‐2801. doi:10.1073/pnas.1104303108 21825174 PMC3287000

[42] Müller P , Rothschild SI , Arnold W , et al. Metastatic spread in patients with non‐small cell lung cancer is associated with a reduced density of tumor‐infiltrating T cells. Cancer Immunol Immunother. 2016;65(1):1‐11. doi:10.1007/s00262-015-1768-3 26541588 PMC11028782

[43] Wang N , Fuh JYH , Dheen ST , Senthil KA . Synthesis methods of functionalized nanoparticles: a review. Biodes Manuf. 2021;4(2):379‐404. doi:10.1007/s42242-020-00106-3

