# Supplementary material for: Living prosthetic breast for promoting tissue regeneration and inhibiting tumor recurrence
Source: Bioeng Transl Med. 2022 Sep 20;8(5):e10409. doi: 10.1002/btm2.10409 (PMC10487338; doi:10.1002/btm2.10409)
Supplement: Supplementary file 1 — Figure S1. Representative TEM images of ZIF Figure S2. Drug release behaviors of U@ZIF‐Gel at pH 7.4 and pH 6.0. The error bars are based on standard errors of the individual samples (n = 3); ANOVA with Tukey's posttest Figure S3. Cell viability of ADSCs cultured with U‐ZIF@Gel and with medium (NC). The error bars are based on standard errors of the individual samples (n = 5); ANOVA with Tukey's posttest Figure S4. Representative H&E stained images of the major organs, heart, liver, spleen, lung, and kidney, in mice treated with saline (Control), urolithin C, and U@ZIF‐Gel Figure S5. Serum levels of ALT and AST in mice treated with U‐ZIF@Gel, control, and urolithin C. The error bars are based on standard errors of the individual samples (n = 5); ANOVA with Tukey's posttest [file BTM2-8-e10409-s001.docx]

**Living Prosthetic Breast for Promoting Tissue Regeneration and Inhibiting Tumor Recurrence**

Wenting Xu ^a,1^, Yu Huang^b,1^, Ho-Yin Yuen^c,1^, Linli Shi^a^, Haiqing Qian^a^, Lijuan Cui^a^, Mengyu Tang^a^, Jiahui Wang^a^, Jie Zhu^a^, Zhirong Wang ^a^, Long Xiao^a,^*, Xin Zhao^c,^**, Lihong Wang^a,^***

^a^Translational Medical Innovation Center, Zhangjiagang Traditional Chinese Medicine Hospital Affiliated to Nanjing University of Chinese Medicine, Zhangjiagang, Jiangsu, 215600, China

^b^Department of Obstetrics and Gynecology, The First People's Hospital of Zhangjiagang, Soochow University, Zhangjiagang, 215600, China

^c^Department of Biomedical Engineering, The Hong Kong Polytechnic University, Hung Hom, Hong Kong, China

Corresponding e-mail address: [zjgfy_spine_xl@njucm.edu.cn](mailto:zjgfy_spine_xl@njucm.edu.cn) (L. Xiao); xin.zhao@polyu.edu.hk (X. Zhao); zjgzyywlh@njucm.edu.cn (L. Wang).

^1^These authors contributed equally to this work





Figure S1. Representative TEM images of ZIF.

Figure S2. Drug release behaviors of U@ZIF-Gel at pH7.4 and pH 6.0. The error bars are based on standard errors of the individual samples (n=3); ANOVA with Tukey’s post-test.

Figure S3. Cell viability of ADSCs cultured with U-ZIF@Gel and with medium (NC). The error bars are based on standard errors of the individual samples (n=5); ANOVA with Tukey’s post-test.


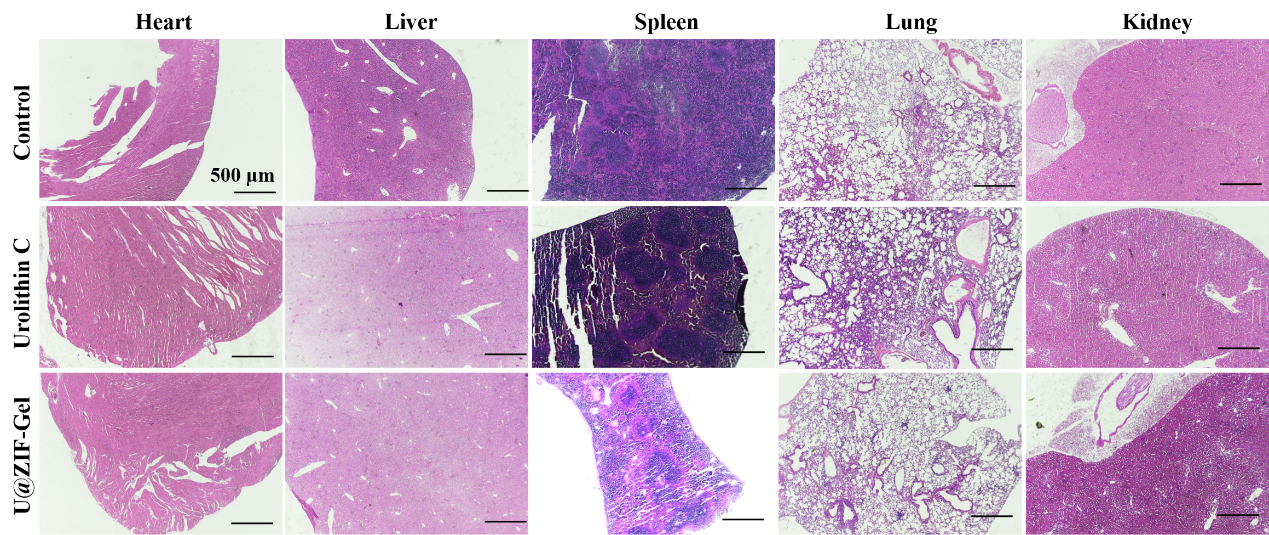


Figure S4. Representative H&E stained images of the major organs, heart, liver, spleen, lung, and kidney, in mice treated with saline (Control), Urolithin C, and U@ZIF-Gel.


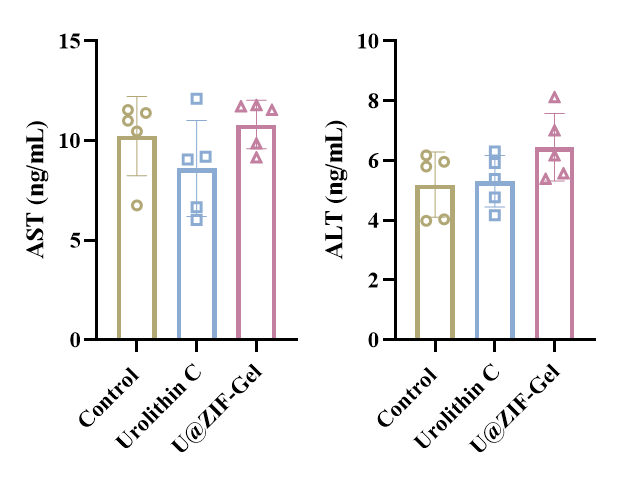


Figure S5. Serum levels of ALT and AST in mice treated with U-ZIF@Gel, Control, and Urolithin C. The error bars are based on standard errors of the individual samples (n=5); ANOVA with Tukey’s post-test.
